# Global Similarities and Multifaceted Differences in the Production of Partner-Specific Referential Pacts by Adults with Autism Spectrum Disorders

**DOI:** 10.3389/fpsyg.2015.01888

**Published:** 2015-12-17

**Authors:** Aparna Nadig, Shivani Seth, Michelle Sasson

**Affiliations:** ^1^School of Communication Sciences and Disorders, McGill UniversityMontreal, QC, Canada; ^2^Centre for Research on Brain, Language and Music, McGill UniversityMontreal, QC, Canada

**Keywords:** autism spectrum disorders, lexical entrainment, referential precedent, referential pact, partner-specificity, common ground, audience design, language production

## Abstract

Over repeated reference conversational partners tend to converge on preferred terms or *referential pacts*. Autism spectrum disorders (ASD) are characterized by pragmatic difficulties that are best captured by less structured tasks. To this end we tested adults with ASD who did not have language or intellectual impairments, and neurotypical comparison participants in a referential communication task. Participants were directors, describing unlexicalized, complex novel stimuli over repeated rounds of interaction. Group comparisons with respect to *referential efficiency* showed that directors with ASD demonstrated typical lexical entrainment: they became faster over repeated rounds and used shortened referential forms. ASD and neurotypical groups did not differ with respect to the number of descriptors they provided or the number of exchanges needed for matchers to identify figures. Despite these similarities the ASD group was slightly slower overall. We examined *partner-specific effects* by manipulating the common ground shared with the matcher. As expected, neurotypical directors maintained referential precedents when speaking to the *same* matcher but not with a *new* matcher. Directors with ASD were qualitatively similar but displayed a less pronounced distinction between matchers. However, significant differences and different patterns of reference emerged over time; neurotypical directors incorporated the *new* matcher's contributions into descriptions, whereas directors with ASD were less likely to do so.

## Introduction

Autism Spectrum Disorders (ASD) are a group of neurodevelopmental disorders or conditions currently defined in the DSM-V by impairments in social communication and interaction alongside the presence of restricted and repetitive patterns of behaviors and interests (American Psychiatric Association, 2013). Perhaps the most noticeable communication difficulties people with ASD experience revolve around initiating and maintaining reciprocal conversation, which requires a host of pragmatic skills (Volden and Lord, 1991; Capps et al., 1998; de Villiers et al., 2007; Nadig et al., 2010). In the 1980s Baron-Cohen and colleagues proposed a compelling explanation for these difficulties, centering on *impaired theory of mind* or the ability to understand other's mental states and understand that these can differ from one's own (Baron-Cohen et al., 1985; Baron-Cohen, 1989). This account was based on early reports of impaired or even “absent” theory of mind in children with ASD and continues to be highly influential, though primarily outside the field of autism (cf. Klin et al., 1992; Frith and Happe, 1994; Mottron et al., 2006, for discussion of the limitations of theory of mind as a comprehensive account of autism).

In psycholinguistics the *impaired theory of mind* account sparked considerable interest in examining pragmatic abilities in ASD, often as a test case for models of reference that are rooted in considerations of *common ground* or the information mutually known to interlocutors (e.g., Clark and Marshall, 1981; Clark and Murphy, 1982; Clark, 1992). In particular, it is often hypothesized that if an aspect of language use is thought to rely on referencing another's mental state (e.g., Does my conversational partner know I call my computer “Titan” or do I need to refer to it as “my computer”?), then people with ASD should not be able to do it in a typical manner, or if they can, then this aspect of language use does not rely on theory of mind. We hope to demonstrate why this all or none approach is overly simplistic and unsubstantiated by current empirical evidence. Consequently a more nuanced view of the use of common ground in ASD is needed to inform models of reference, just as a multifaceted view has evolved on the use (Brown-Schmidt and Hanna, 2011) and representation (Brown-Schmidt, 2012) of common ground in the neurotypical population.

Although the impaired theory of mind account and conventional expectations hold that people with ASD should categorically lack sensitivity to common ground, research exploring whether people with ASD are sensitive to their conversational partner's perspective paints a more complex and gradient picture. Nadig et al. (2009) found that half of the children with ASD they tested showed reduced sensitivity to a partner's visual perspective when producing descriptions for objects that were either shared in common ground or visible only to them, as is commonly expected. However, the other half of children with ASD, who had higher formal language abilities, were indistinguishable from their typical peers with respect to reliance on common ground in this structured task. In a narrative task, DE Marchena and Eigsti (2016) manipulated common ground by having the listener share prior exposure (or not) to cartoon clips that were later narrated by participants. Their sample of adolescents with ASD showed sensitivity to common ground, communicating differently in its presence on a number of measures (explicit references of common ground, disfluencies, and independent ratings of communicative quality). Yet, while typically-developing adolescents showed a standard referential shortening effect, producing fewer words in narratives for listeners who shared exposure relative to those who did not, adolescents with ASD did not show this effect as a group. However, older ASD participants and those with better social skills performed similarly to their typical peers on this more open ended task. Taken together these findings demonstrate that reliance on common ground by children and adolescents with ASD is best viewed as delayed rather than absent, and that there is significant variation among people with ASD. Many speakers with ASD (who do not have language or intellectual impairment, as in these studies) are aware of differences in their partner's perspective, but are less adroit in addressing discrepancies in common ground in their spontaneous language use.

To date, one study has examined the negotiation of discrepant common ground in adults with ASD. Begeer et al. (2010) examined sensitivity to a partner's visual perspective during the comprehension of referential descriptions (employing a task similar to that used by Nadig et al., 2009) and found nearly identical performance between adults with and without ASD. Given the findings from children and adolescents reported above this is not surprising, as sensitivity to common ground increases with age and/or formal language abilities in ASD. Finally, Slocombe et al. (2013) used interactive tasks to investigate the alignment of lexical choice, spatial frame, and syntactic structure in adults with ASD without directly manipulating common ground information. They hypothesized lexical choice would involve *audience design* (Clark and Marshall, 1981) or the tailoring of language to the knowledge or competence level of a conversational partner to promote successful communication (e.g., Bortfeld and Brennan, 1997; Branigan et al., 2011). To examine lexical choice, Slocombe and colleagues used a referential communication task where a confederate described familiar pictures using rare names (e.g., chapel rather than church), and then measured whether participants would “align” with this uncommon name when later referring to the same picture. Contrary to the authors' predictions, they found that adults with ASD (specifically Asperger's Disorder using DSM-IV criteria) were as likely as comparison participants to use the uncommon name. They also aligned with their conversational partners with respect to syntactic structure and spatial frame of reference (Slocombe et al., 2013). In interpreting these findings, both Begeer et al. (2010) and Slocombe et al. (2013) highlight that the lack of group differences in their studies was likely due to the nature of the tasks employed, which were highly structured and goal-directed. There are a number of reasons why performance would be enhanced in structured language tasks vs. communication in real life. For one, the interaction is more predictable and the problem space is limited, so it may become easier to incorporate contextual information including a partner's perspective (Nadig et al., 2009). Begeer and colleagues proposed that over arousal and a focus on local rather than global processing that is observed in many individuals with ASD could be “neutralized in structured social interaction” (Begeer et al., 2010, p. 115). Importantly these authors (Nadig et al., 2009; Begeer et al., 2010; Slocombe et al., 2013) emphasize that audience-design effects from structured tasks are no less valid as evidence of reliance common ground during communication, but rather that structured tasks alleviate other factors and task demands that may normally interfere with the effective use of common ground in people with ASD. Nevertheless, these findings suggest that more open-ended tasks are required to capture difficulties with audience design that are commonly encountered by adults with ASD in daily life.

The goal of the current study was two-fold. First, we examined referential efficiency in the evolution of referential descriptions over multiple rounds of a communication game where directors, adults with or without ASD matched on verbal IQ, refer to novel tangram images (geometric shapes that are difficult to describe) so that matchers can identify them from an array of other tangrams (e.g., Krauss and Weinheimer, 1964, 1966; Clark and Wilkes-Gibbs, 1986; Krauss, 1987; Schober and Clark, 1989). We know from prior work using similar methods that referential efficiency improves over time through a process of *lexical entrainment* (Garrod and Anderson, 1987) where a preferred lexical form or *referential pact* (Brennan and Clark, 1996) is collaboratively agreed upon through proposals from the director as well as back channel responses from the matcher. To our knowledge the collaborative construction of novel referential terms has not previously been investigated in ASD. Though such a communication game is structured by definition, we view ours as a more open-ended task than those used previously due to a combination of factors: we examine the participant's open-ended production rather than comprehension of scripted instructions, stimuli is novel and unlexicalized, consequently it is difficult to describe and there is no closed set of options to choose from (e.g., common vs. rare), we investigate the evolution of descriptions over repeated rounds of reference, and finally we include an experimental manipulation (described below) to assess the impact of a change in partner, disturbing the structure that had been established. To examine participants' ability to entrain over time we analyze the duration of repeated rounds of the game with the same set of stimuli. We also investigate the number of descriptors (defined in Section Data Coding) directors produce when describing tangram stimuli, as well as the number of exchanges between director and matcher until the matcher is able to identify the target referent. Speakers with ASD are known to have difficulty providing the appropriate level of information for a given communicative situation, being over- or under-informative in different circumstances (cf. Volden et al., 1997; Dahlgren and Sandberg, 2008; Nadig et al., 2009), and to be stereotyped in their language use (Philofsky et al., 2007), which may make them less efficient in this collaborative task. However, previous data from adults with ASD without intellectual or language impairment, similar to our sample, demonstrates that lexical alignment is intact in this group (Slocombe et al., 2013). Therefore, we predicted few if any differences on measures of referential efficiency.

Second, we investigated the partner-specificity of any *referential pacts* established by manipulating the experience and thus the common ground shared with the matcher. Critically, in interactive settings (cf. Brown-Schmidt, 2009) lexical entrainment has been shown to be partner-specific. After conversational partners develop a referential pact, if a new partner who was not involved in entrainment is introduced, the entrained term is less likely to be maintained by directors (Brennan and Clark, 1996) or expected by matchers (Metzing and Brennan, 2003; Brown-Schmidt, 2009). Recent work with typically-developing children shows that children as young as 4 years old maintain referential precedents with their peers in a partner–specific manner (Köymen et al., 2014) and that 3- and 4-year-olds expect adult speakers to maintain referential precedents in a partner–specific manner (Matthews et al., 2010; Graham et al., 2014). The mechanisms underlying the comprehension of referential precedents is an area of active debate, at the heart of which is whether high-level common ground inferences or low-level memory mechanisms (episodic priming and encoding cues) best explain the effects (Brennan and Hanna, 2009; Brown-Schmidt, 2009; Shintel and Keysar, 2009; Kronmüller and Barr, 2015). The task we use stands somewhat outside this debate as it is a production task, and prior work on production used a different paradigm with familiar objects with known names rather than tangram stimuli (Brennan and Clark, 1996; Köymen et al., 2014). We view our task, where participants as directors need to create agreed upon terms for complex novel stimuli through interaction with a matcher, as one that inherently requires collaboration. Therefore, if partner-specific effects are found, they are likely to follow from considerations of whether a referential precedent is shared in common ground with a specific matcher or not, a point that will be returned to in the discussion.

We analyzed partner-specific effects by comparing expected differences across conditions in the duration of Round 1 vs. Round 4, where the new matcher was introduced in the *new* condition but the same matcher continued the game in the *same* condition. For a more precise measure of how directors may adapt descriptions to a *new* matcher, we examined the maintenance of the referential precedent from the prior round on critical Round 4 in the *same* vs. *new* conditions, as well as how they continued to interact with the matcher on Round 5, the end of the game with a given set of cards. Finally we explored whether these two variables were related on critical Round 4: Is the duration difference, which was expected to be a delay in the presence of a *new* matcher, related to whether directors continued to maintain the referential precedent or not? We predicted that neurotypical directors would maintain pacts with the same matcher but elaborate on the referential precedent or chose a different term when speaking to a new matcher, consistent with prior findings. When it comes to the ASD group, a staunch impaired theory of mind account would predict that they would show no difference between same and new matcher conditions, continuing to use the same descriptions regardless of differences in the common ground shared with their listener. However, given the findings reviewed above showing basic sensitivity to discrepancies in common ground in ASD, we expect this group to show some sensitivity to the change in partner but in a less pronounced way than the neurotypical group.

Finally, to obtain a direct measure of the collaborative nature of lexical entrainment (Clark and Wilkes-Gibbs, 1986) we examined how likely directors were to incorporate the matcher's proposals (when provided) for how to describe the figure. We predicted that matchers would suggest more descriptors on early rounds of discussing a figure, before a referential pact was established. We expected that participants with ASD may be less likely to engage in this collaborative behavior.

## Materials and methods

### Participants

Thirteen adults with ASD and 13 neurotypical adults (NT; from the general population with no known developmental disorders) were included in the sample. An additional 4 ASD participants were tested, but no video record of their sessions was available for analysis due to experimenter error. An additional 18 NT participants were tested but only those who could be closely matched to each of the ASD participants are included here. Participants with ASD were participating in a larger transition support service for young adults with ASD and were recruited through advertisements posted at local autism organizations, college offices for students with disabilities, and social service providers. The NT comparison participated in a longer 2 h testing protocol including the referential communication task presented here. They were recruited either through a psychology department subject pool, receiving partial course credit for participation, or through word of mouth and advertisements in the community, receiving $10 for participation. This study received ethics approval from the University of McGill Faculty of Medicine Institutional Review Board. All participants gave written informed consent in accordance with the Declaration of Helsinki.

Participants ranged from 18 to 29 years old; age did not differ significantly different between groups [ASD: *M* = 22 years 2 months, *SD* = 4 years 2 months, NT: *M* = 21 years 2 months, *SD* = 11 months, *t*_(1, 24)_ = 0.90, *p* = 0.38, *r* = 0.17]. Gender proportion was also similar across groups (ASD: 7 males, 6 females, NT: 5 males, 8 females, χ^2^ = 0.62, *p* = 0.43, φ = 0.15). To ensure the groups had similar verbal abilities, allowing for comparison of pragmatic abilities specifically on the referential communication task, they were administered the verbal subtests (Vocabulary and Similarities) of the Wechsler Abbreviated Sale of Intelligence (WASI; Wechsler, 2008) to obtain a measure of verbal IQ. Groups did not differ significantly with respect to verbal IQ [ASD: *M* = 113, *SD* = 10, NT: *M* = 115, *SD* = 9, *t*_(1, 24)_ = 0.53, *p* = 0.60, *r* = 0.10]. All but two participants (one from each group) were native speakers of English. Those who were not native speakers had been using English their daily life for 10 years or more and had completed secondary or university education in English, moreover they scored in the average range or higher on an English test of verbal IQ.

Community diagnoses of ASD were confirmed in our study by administration of the ADOS-2 module 4 (Lord et al., 2012), using the revised algorithm for module 4 (Hus and Lord, 2014) or, when possible, parent report of early autism symptoms using the Social Communication Questionnaire (SCQ, Rutter et al., 2003). Nine of 13 ASD participants met ASD criteria (i.e., scores of 8 or higher, *M* = 13, range = 8–23) on the ADOS-2 based on current functioning and the remaining four participants met criteria for ASD based on their early development, as reported by their parent on the SCQ (i.e., scores of 15 or higher), but fell short of meeting ADOS-2 criteria based on current functioning (having ADOS-2 scores from 5 to 7). Prior to the lab visit, participants in the NT group completed a demographic questionnaire asking if they had ever been diagnosed with a developmental disorder, and whether they had a first or second degree relative with ASD; potential NT participants meeting either of these criteria were excluded. Of potential NT participants who completed the questionnaire, two were excluded from participation.

### Materials

**Figure 1:**
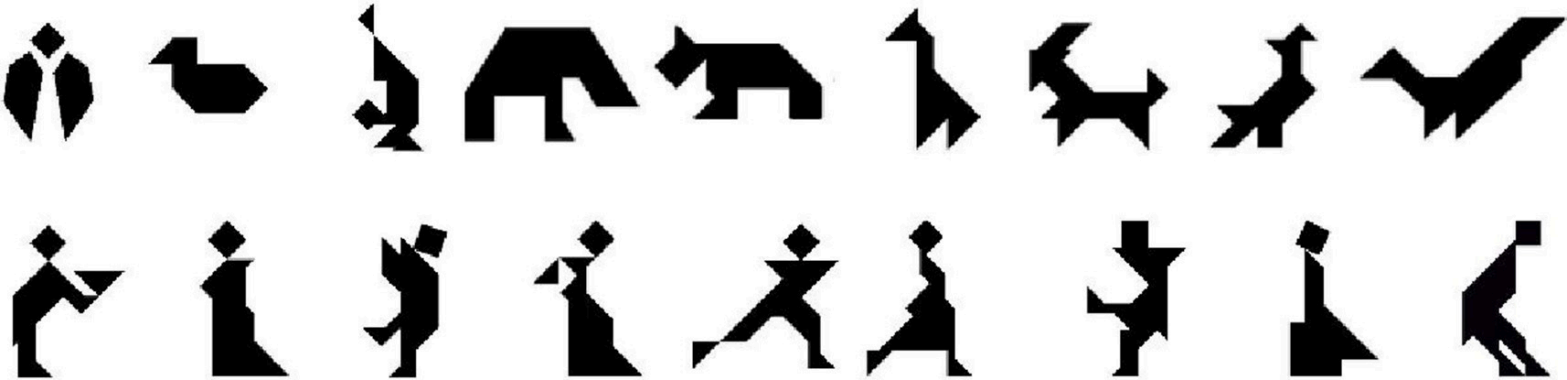
**Tangram stimuli used in our task, Set A (top row) and Set B (bottom row)**. Each set was used for one condition (*same vs. new* matcher).

Eighteen tangram figures were printed in black ink on white cardstock. Two sets of nine stimuli were used, one set resembled animals (Set A), and the second resembled people (Set B), see Figure 1. Cards were laminated and two copies were made of each card to have identical sets for the director and matcher. Two easel boards with a 3 by 3 numbered grid marked on them were used to present the stimuli. Velcro in each square of the grid and on the back of each card allowed the cards to be attached and removed from the easel.

### Procedure

We employed a collaborative referential communication game to assess production, incorporating elements from two previous lines of research: spontaneous lexical entrainment while describing complex novel stimuli (Krauss and Glucksberg, 1969; Clark and Wilkes-Gibbs, 1986), and manipulation of the *same* vs. *new* partner when studying the use of referential precedents in interactive tasks (Brennan and Clark, 1996, Experiment 3; Metzing and Brennan, 2003; Brown-Schmidt, 2009).

Participants always played the role of director, describing tangram stimuli to an experimental confederate who acted as the matcher. To measure partner specific effects two different matchers (original or *same* and *new*) were introduced in the *new* matcher condition, details provided below. An experimenter who conducted the longer testing session introduced the participant to the *same* matcher, who was presented as a lab member who had been called to help with this particular task. The experimenter explained the task to the director and the *same* matcher concurrently, assuming no familiarity with the task. Matchers were undergraduate or graduate student research assistants, or in rare cases a faculty member if assistants were not available to fill all roles. For the sample reported here, in total 8 confederates played the role of the *same* matcher (median number of times playing this role = 4) and 10 confederates played the role of the *new* matcher (median number of times playing this role = 2.5). Matchers were instructed to respond naturally in the game and to ask for more information as required to complete the task. As the majority of matchers played the task only a few times over many months, and that the initial descriptions for each figure varied greatly across directors, the stimuli remained relatively new to them.

The director and matcher were seated across from each other at a table, each with a large easel in front of them so that neither could see the other person nor what was displayed on his/her easel. The experimenter (E), who conducted the longer session, introduced the task and how it was played, and was responsible for placing and removing stimuli cards and operating a videocamera. Before each round, E placed nine cards on the director's easel in a random order. E explained to both the director and matcher that she would put nine cards up on the easel, and that the matcher had an identical set of cards on the table in front of her. The director's task was to describe the cards in sequence (squares 1–9 were indicated on the board) so the matcher could place her cards to match the director's display. Three practice cards were used to familiarize dyads with the task. During the practice round it was reinforced that the director should move in order from square one to nine, that the matcher could ask questions at any time for clarification, and that the matcher should say “okay” or “got it” when she located the correct card as the director would not be able to see this.

Ten rounds of this game were played in total: five rounds with a given set of cards for each of the two experimental conditions (*same* matcher vs. *new* matcher). The director described nine cards, in sequence, on each round. At the end of the round, E shuffled the cards and placed them up on the director's easel in a random order, starting the next round.

The matcher's knowledge of referential precedents was manipulated as follows. In the *same* matcher condition, the director described the cards to the *same* matcher for three rounds. At this point the matcher said she forgot to tell her friend something next door and left. She returned after a minute, the game continued for rounds 4 and 5 with the *same* matcher.

In the *new* matcher condition the director also played the game with the *same* matcher for rounds 1–3. However, this time the *same* matcher said that she really needed to go to the bathroom and that her friend could step in for her. The *same* matcher left and the *new* matcher came in a minute later. E quickly introduced the *new* matcher to the game and its rules, and rounds 4 and 5 were played with the *new* matcher. Thus, the *new* matcher was also presented as a lab member, but one who was naïve to the game; the Experimenter introduced the game to this *new* matcher as if she had no prior knowledge of it.

A different set of cards (A or B in Figure 1) was used for each condition. Card set and order of condition were counterbalanced within each group by assigning each subsequent participant tested to one of four orders (e.g., Set A or Set B first, *same* or *new* condition first). Given an uneven sample size (13 in each group) and that more participants were tested than those included in the final sample, this resulted in card set and condition not being fully balanced. In the *same* matcher condition 8/13ASD participants and 7/13 NT participants received Set A, with the remaining receiving Set B; the opposite card set was used in the *new* matcher condition. For both ASD and NT groups 5/13 participants had the *same* matcher condition first, the remaining 8 had the *new* matcher condition first.

### Data coding

Data was transcribed from video recordings of the task. The *duration of each round* was recorded while transcribing. Tangram descriptions were divided into descriptors, defined as any noun or modifier describing the figure as a whole. Adjectives modifying a part of the figure were not counted as their own descriptor. For example, “the skater with his left leg stretched out and a diamond head” was coded as three *initial descriptors*: skater; left leg stretched out; diamond head. In this case, left was not considered a separate descriptor because it describes leg and not the skater. The *number of exchanges* or turns between director and matcher when working on a figure, from the initial description until the matcher located the card, were also coded. An exchange was defined as one description by the director, and one verbal response by the matcher. The response by the matcher could be a question or statement, or a confirmation (“Okay, got it” or “uh huh,” participants were told during practice that they should confirm in this fashion since their partner could not see when he/she found the card).

*Relation to the referential precedent of the prior round* was coded into one of four categories. In doing so, determiners, prepositions and other function words were excluded when determining informational equivalence; thus “the lady who is walking” was considered equivalent to “walking lady” (Brennan et al., 2013). The *same* category was used when two descriptions were informationally equivalent. *Same-simplified* refers to descriptions that maintained the conceptualization of the previous round, but used fewer descriptors as is typical in lexical entrainment, apparently because fewer were needed when a referential pact had been established. For instance:

Round 1: Director: The waiter with the triangle tray, facing right
Round 2: D: The waiter with the tray
Round 3: D: The waiter
Round 4: D: Waiter
Round 5: D: Waiter

In this example the description categories on Rounds 2 and 3 (with respect to the prior round) were *same-simplified* and description categories for Rounds 4 and 5 were same.

Sometimes directors would use the same conceptualization but add additional information or descriptors. This was coded as *same-expanded*. For example:

Round 1: D: The sad dog
Round 2: D: The sad dog, facing left

Occasionally the director would offer a conceptualizations that was completely different from the prior round, these were coded as *different*.

Round 1: D: The bird facing left with two triangle feet
Round 2: D: The giraffe

*Incorporation of matcher's descriptors* was coded as direct measure of the collaborative nature of lexical entrainment (Clark and Wilkes-Gibbs, 1986). For each of the 9 cards described and entrained upon in each condition (*same* or *new* matcher), how the director handled the matcher's spontaneous contributions regarding how to describe the figure were coded as follows: *yes* (matcher's descriptor incorporated by director on a subsequent round), *no* (no incorporation of descriptors suggested by matcher), or *N/A* (matcher did not propose any descriptors). This code was assigned at two time points for each card: for exchanges through the end of Round 3 (which always involved the *same* matcher in both conditions), and again for those from Round 4 through 5 (which involved a change in matcher in the *new* matcher condition). For instance, the following exchange in the *new* matcher condition received the code of *yes* for the Round 4 through 5 incorporation variable. It should be noted that this variable is likely affected by unmeasured differences with respect to the matcher's contributions (e.g., how plausible they were as descriptors, whether they offered a contrasting conceptualization or followed the director's conceptualization), since many confederates played the role of matcher and their only direction was to respond naturally to complete the task.

Participant 117
**Round 4**
  **Director:** a four legged or two legged animal facing the right The head is a parallelogram and its back leg is a rectangle and the front legs look like paws
  ***New* Matcher:** does it look like an elephant if the parallelogram is a trunk?
  **Director:** yeah, it does look like an elephant
**Round 5**
  **Director:** an elephant facing right

#### Coding reliability

A coding system was developed by the authors over multiple iterations of trying to capture the construct of referential precedent in the current production corpus involving the description of complex novel stimuli (as opposed to familiar basic and subordinate level terms, e.g., shoe and penny loafer, Brennan and Clark, 1996). The second author trained two additional undergraduate students, who were blind to the design and hypotheses of the study as well as group membership, on the final coding system via discussions and work on two training files until they reached consensus in their coding. Across variables, 20–33% of the participants in each group were double coded to calculate inter-coder reliability. For *number of initial descriptors*, correlations indicated very high agreement between both additional coders (*r* = 0.96 and 0.99) and the second author. *Number of exchanges* was calculated by an excel formula based on the cells where each director description and matcher response or question were entered in sequence. Reliability on referential *precedent categories* was measured by Cohen's unweighted kappa, which was reasonably high between each of the additional coders (Kappa = 0.90 and 0.78) and the second author. Finally, reliability for *Incorporation of matcher's descriptors* was obtained between the third author and an undergraduate student blind to study hypotheses and group membership. Cohen's unweighted kappa a was very high for incorporation by Round 3 (Kappa = 0.92) as well as by Round 5 (Kappa = 0.98).

## Results

Effect size is provided for each contrast using *r* for pairwise comparisons, for which a small effect is defined as 0.1, a medium effect is 0.3 and a large effect is 0.5, and with partial eta squared ${\text{η}}_{p}^{2}$ for ANOVA effects, which reflects the portion of unique variance on the dependent variable that is explained by the independent variable.

### Referential efficiency^1^

#### Round duration

**Figure 2:**
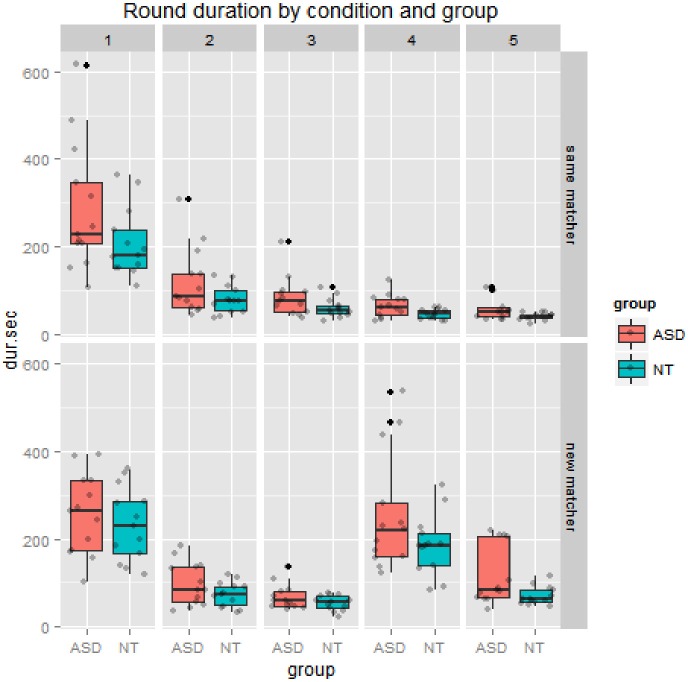
**The top panel shows the same matcher condition, where dyads in both groups get much faster over 5 rounds of discussing the figures, reflecting lexical entrainment. Results differ however in the *new* matcher condition in the bottom panel, where dyads in both groups show a disruption of lexical entrainment when a new matcher is introduced on the fourth round. Gray dots indicate jittered data points, black dots indicate outliers**.

For a global analysis of whether lexical entrainment took place over the 5 rounds of each condition, we submitted data on round duration in seconds to a mixed ANOVA with round (1 through 5) and condition (*same* or *new* matcher) as within-subjects variables and group (ASD vs. NT) as a between subjects variable. As seen in Figure 2, there were strong main effects of round, *F*_(4, 96)_ = 75.12, *p* < 0.001, ${\text{η}}_{p}^{2}=0.76$, and of condition, *F*_(1, 24)_ = 14.15, *p* = 0.001, ${\text{η}}_{p}^{2}=0.37$. There was also a significant effect of group, *F*_(1, 24)_ = 7.76, *p* = 0.01, ${\text{η}}_{p}^{2}=0.24$. This was due to the ASD group having a higher average round duration (141 s) relative to the NT group (105 s) with a small effect size, *t*_(1, 234.12)_ = 2.89, *p* < 0.001, *r* = 0.18. There was a significant interaction between round and condition, *F*_(4, 96)_ = 27.10, *p* < 0.001, ${\text{η}}_{p}^{2}=0.53$. No other interactions were significant.

Remaining variables were averaged over the rounds of each condition and were analyzed using mixed repeated-measures ANOVAs with condition (*same* or *new* matcher) as a within-subjects factor and group (ASD, NT) as a between subjects factor.

#### Initial number of descriptors per figure

There was a significant main effect of condition (*same* vs. *new* matcher), *F*_(1, 24)_ = 9.34, *p* = 0.005, ${\text{η}}_{p}^{2}=0.28$. There was not a main effect of group, *F*_(1, 24)_ = 0.49, *p* = 0.51, ${\text{η}}_{p}^{2}=0.02$. Finally, there was no interaction between group and condition, *F*_(1, 24)_ = 0.18, *p* = 0.70, ${\text{η}}_{p}^{2}=0.01$. The NT group increased from a mean of 2.15 descriptors when speaking to the *same* matcher to 2.56 descriptors when speaking to the *new* matcher. The ASD group also increased across conditions, from a mean of 2.22 descriptors when speaking to the *same* matcher to 2.74 descriptors with the *new* matcher.

#### Number of exchanges per round

**Figure 3:**
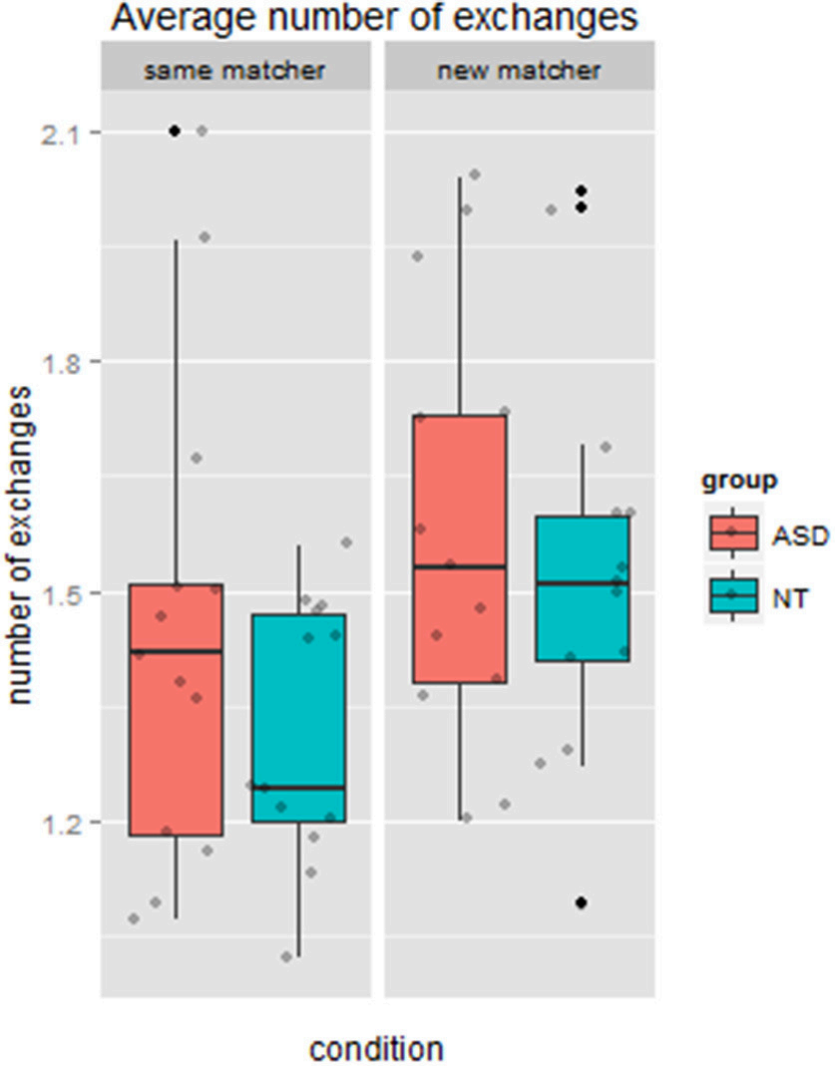
**Average number of exchanges required for the matcher to locate the figure, by condition and group**.

There was a significant main effect of condition (*same* vs. *new* matcher), *F*_(1, 24)_ = 11.13, *p* = 0.003, ${\text{η}}_{p}^{2}=0.32$. Again there was no a main effect of group, *F*_(1, 24)_ = 1.13, *p* = 0.30, ${\text{η}}_{p}^{2}\;=0.05$. Finally, there was no interaction between group and condition, *F*_(1, 24)_ = 0.60, *p* = 0.45, ${\text{η}}_{p}^{2}=0.02$. Figure 3 shows that the NT group increased from a mean of 1.32 turns when speaking to the *same* matcher to 1.53 turns when speaking to the *new* matcher. The ASD group also increased, from a mean of 1.45 turns with the *same* matcher to 1.59 turns with the *new* matcher.

### Partner-specific adaptation

Round 4 was the critical point in the experiment; it was the first round after the original matcher left the room momentarily and returned in the *same* condition, or was replaced in the *new* matcher condition. We predicted that audience design effects would be seen most prominently at this point in the NT group. We predicted the ASD group would respond in a qualitatively similar way, showing some sensitivity to the change in matcher, but that they would smaller differences between the *same* and *new* matcher conditions than the neurotypical group.

#### Difference in duration of round 1 vs. 4

**Figure 4:**
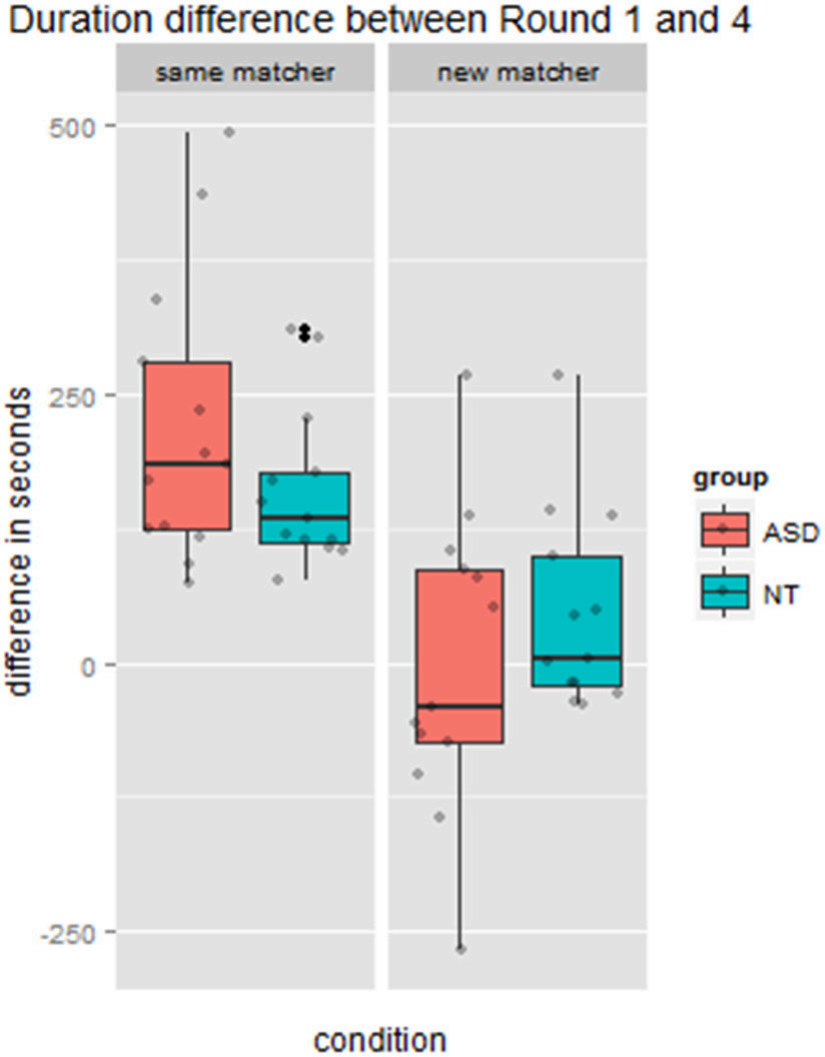
**Difference in duration of Round 1 vs. Round 4, where the new matcher switch occurred, by condition and group**. Positive values indicate speeding up over four rounds of referring to the same figures, and a 0 duration difference indicates taking the same time on Round 4 as on the first presentation of the cards on Round 1.

Through the process of lexical entrainment conversational partners typically speed up over repeated references to the same entity. To measure the extent to which the *new* matcher disrupted this process, controlling for baseline description speed, we calculated a difference score: duration of Round 1 minus the duration of Round 4, which is positive when dyads get faster over time. As would be expected from Figure 2 above, there was a significant main effect of condition (*same* vs. *new* matcher), *F*_(1, 24)_ = 38.48, *p* < 0.001, ${\text{η}}_{p}^{2}=0.62$. As for other variables, there was no main effect of group *F*_(1, 24)_ = 0.02, *p* = 0.88, ${\text{η}}_{p}^{2}=0.00$. However, there was a marginally significant interaction between group and condition, *F*_(1, 24)_ = 3.88, *p* = 0.06, ${\text{η}}_{p}^{2}=0.14$. The NT group went from a mean decrease of 162 s when speaking to the *same* matcher to only 46 s when speaking to the *new* matcher. The ASD group went from a mean decrease of 221 s when speaking with the *same* matcher to an *increase* of 2 s with the *new* matcher. This pattern is depicted in Figure 4.

#### Maintenance of referential pact on round 4

**Figure 5:**
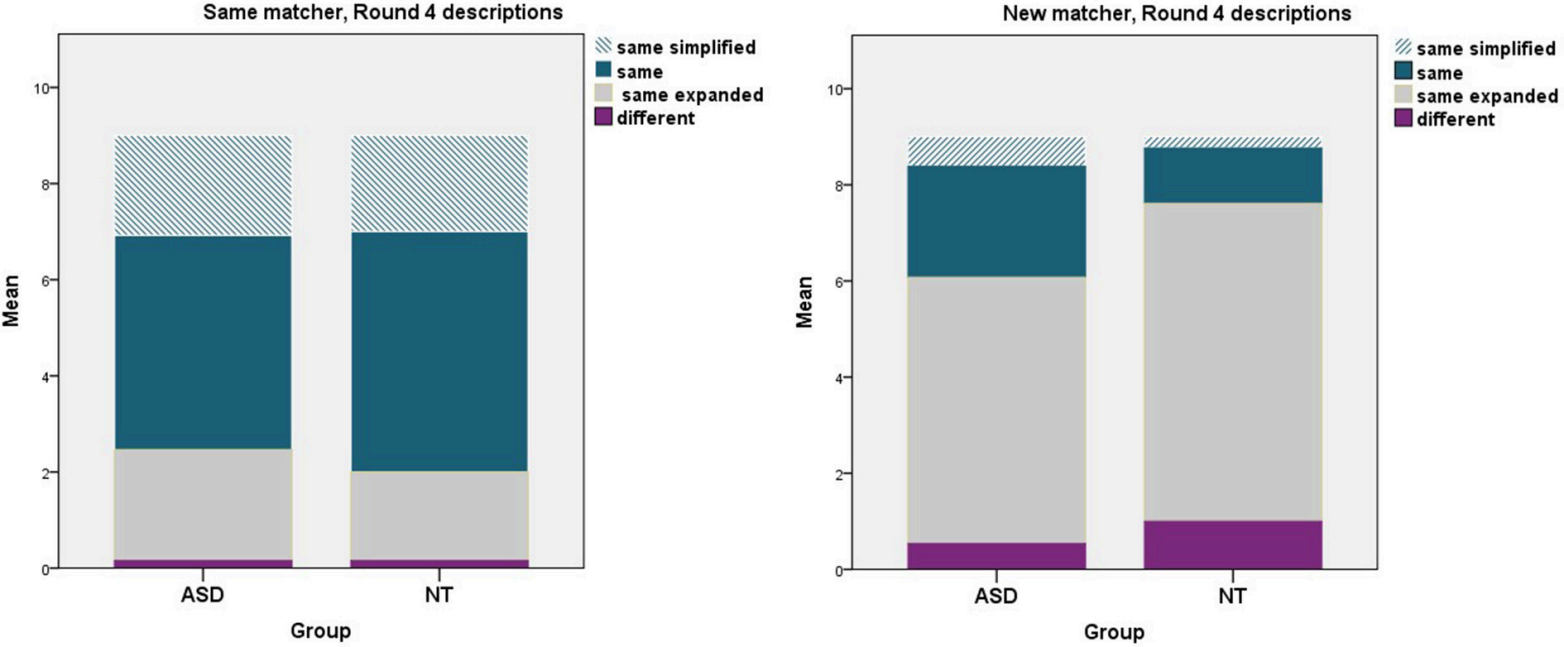
**Types of descriptions, with respect to the referential precedent of the prior round, given on critical Round 4 to the *same* matcher (left) vs. *new* matcher (right)**.

We predicted that on Round 4 directors in both groups would maintain the referential pact they had been using with the *same* matcher, that is, repeat the referential precedent or use a reduced form of it. For the *new* matcher we predicted that the NT group would spontaneously engage in audience design for the *new* matcher, who did not share knowledge of the referential precedent, by elaborating on it or using a different lexicalization. Finally, we predicted the ASD group would show less sensitivity to the *new* matcher by being more likely to maintain the referential pact they had established with the original matcher. Figure 5 shows a complete tally of the types of descriptions given on critical Round 4 in relation to Round 3 descriptions.

**Figure 6:**
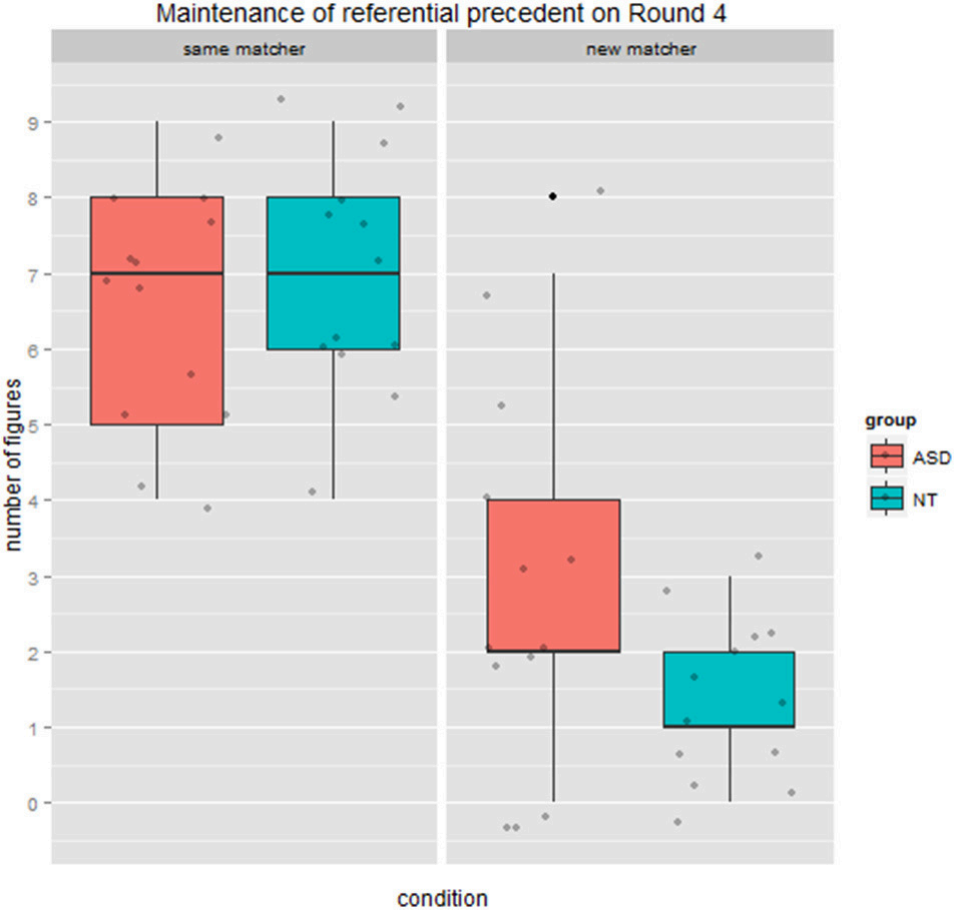
**Maintenance of referential precedent from Round 3 on Round 4, by condition and group**.

Our analysis focused on maintenance of the referential precedent (including same-simplified and same descriptions). There was a significant main effect of condition (*same* vs. *new* matcher), *F*_(1, 24)_ = 68.46, *p* < 0.001, ${\text{η}}_{p}^{2}=0.74$. Once again there was no main effect of group, *F*_(1, 24)_ = 1.61, *p* = 0.22, ${\text{η}}_{p}^{2}=0.06$. There was, however, a marginally significant interaction between group and condition, *F*_(1, 24)_ = 3.21, *p* = 0.08, ${\text{η}}_{p}^{2}=0.12$. We followed this up with a planned comparison between groups in the *new* matcher condition specifically. In line with our prediction, the ASD group were marginally more likely to maintain the referential pact than the NT group, with a medium effect size, *t*_(1, 24)_ = 2.03, *p* = 0.05, *r* = 0.37. At the individual level, all 13 NT directors maintained 3 or fewer referential precedents on Round 4 with the *new* matcher, while 9/13 directors with ASD displayed the same pattern, but the remaining 4 maintained 4–8 referential precedents. As seen in Figure 6 the NT group showed an extreme difference between conditions, maintaining referential precedents for a mean of 7 out of 9 figures when speaking to the *same* matcher, but only for 1.38 figures when speaking to a *new* matcher. The ASD group was less pronounced in this distinction, decreasing from a mean of 6.53 referential precedents maintained with the *same* matcher to 2.92 maintained with *new* matchers.

**Figure 7:**
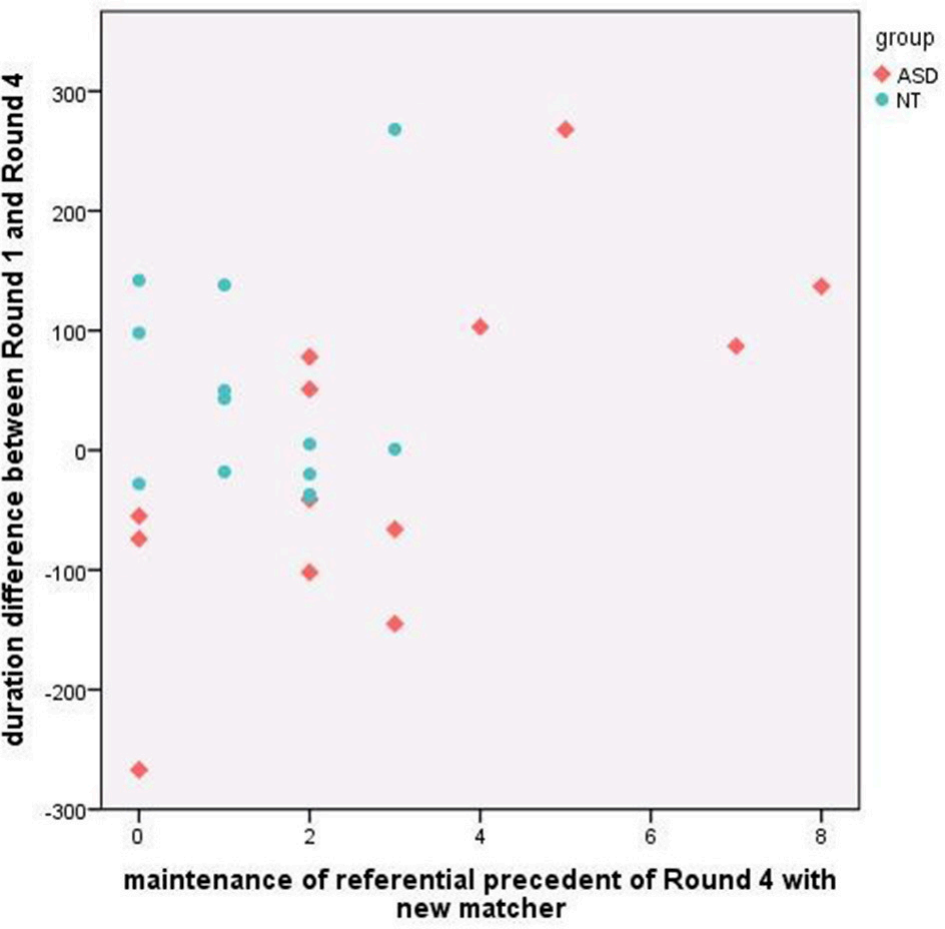
**Relation between maintenance of referential precedent on Round 4 with new matcher and the duration difference between Round 1 and Round 4** (positive values indicate speeding up over repeated reference).

We also examined if Round 1 minus Round 4 duration difference was related to maintaining the referential pact on Round 4 in the *new* matcher condition specifically. We reasoned that that *maintaining the referential precedent* on this round may *slow the dyad's interaction*, since the *new* matcher lacked knowledge of the referential precedent. Consequently we expected that greater maintenance of referential precedents would be inversely related to duration difference (where positive values indicate speeding up). Results are shown in Figure 7. The correlation in the NT group was in the direction of our prediction but did not reach significance (*r* = −0.20, *p* = 0.37), likely because there was little variation in maintaining the precedent when speaking to the *new* matcher. There was a significant correlation in the ASD group (*r* = 0.52, *p* = 0.02), but in the opposite direction of our prediction. In fact, in cases where ASD participants maintained more precedents with the *new* matcher, dyads got through Round 4 more quickly. Conversely, in cases where ASD directors tended not to maintain precedents with the *new* matcher, as NT directors did, dyads actually took longer to complete Round 4. Our interpretation of this finding is that it took ASD directors more time to adapt to the *new* matcher in the manner that NT directors did (by elaborating on the referential precedent or using a different term). It is also possible that the *new* matchers' responses contributed to this longer duration, however, since the *new matcher* had just started playing the game, and there were no group differences in referential efficiency measures on the part of the director, it is unlikely that matchers had a basis on which to respond differently to ASD vs. NT directors.

#### Maintenance of referential pact on round 5

**Figure 8:**
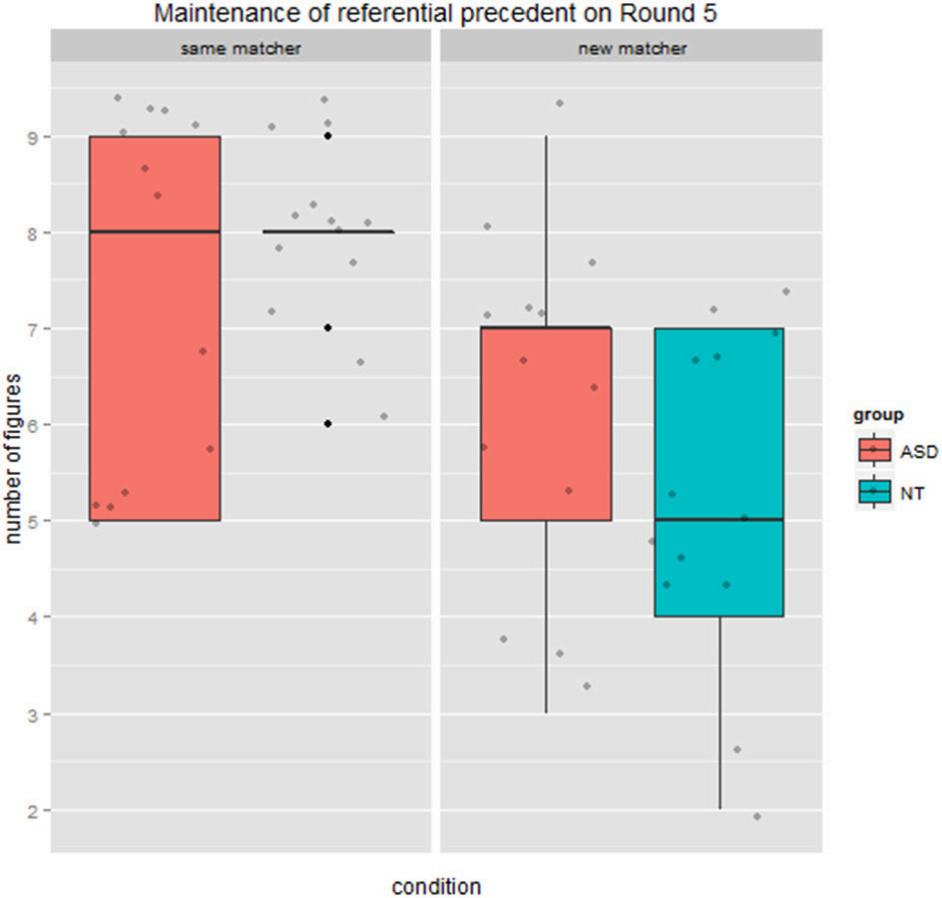
**Maintenance of referential precedent from Round 4 on Round 5, by condition and group**. Note: The NT group displayed little variability, with an interquartile range of 1 that was too small to appear in this boxplot.

To examine how entrainment would unfold in the presence of the *new* matcher we also examined maintenance of referential pacts from Round 4 on Round 5. There was a significant main effect of condition (*same* vs. *new* matcher) *F*_(1, 24)_ = 27.13, *p* < 0.001, ${\text{η}}_{p}^{2}=0.53$. Again there was no main effect of group, *F*_(1, 24)_ = 0.14, *p* = 0.71, ${\text{η}}_{p}^{2}=0.01$. Importantly, there was a significant interaction between group and condition, *F*_(1, 24)_ = 4.98, *p* = 0.03, ${\text{η}}_{p}^{2}=0.17$. Figure 8 shows that the NT group maintained their referential precedent on the last round of the game for a mean of 7.92 of 9 figures with the *same* matcher, but for 5.23 figures when speaking to a *new* matcher. The ASD group was less divergent between conditions, decreasing from a mean of 7.30 referential precedents maintained with the *same* matcher to 6.23 maintained with *new* matchers.

#### Incorporation of matcher descriptors

This was a direct measure of how collaborative entrainment was, that is, whether directors incorporated descriptors suggested by the matcher on a subsequent round. Results are provided in Table 1 below. The majority of data was missing for the *same* matcher condition, Round 4 through 5 because an entrained term was generally set and the *same* matcher tended not to suggest descriptors at this point, giving no opportunity for incorporation. Given this, analyses focused on the *new* matcher condition, which reflects partner-specific changes. A mixed ANOVA was conducted with subjects factor of time point (by end of Round 3, Round 4 through 5) and the between subjects factor of group. The effect of time point was not significant *F*_(1, 22)_ = 1.83, *p* = 0.19, ${\text{η}}_{p}^{2}=0.08$. There was however a significant main effect of group, *F*_(1, 22)_ = 4.97, *p* = 0.04, ${\text{η}}_{p}^{2}=0.18$. This was due to the ASD group having a reduced tendency to incorporate the matcher's contributions (0.31) relative to the NT group (0.48). There was also a marginal interaction between group and time point, *F*_(1, 22)_ = 3.92, *p* = 0.06, ${\text{η}}_{p}^{2}=0.15$. As seen in Table 1, this reflected the fact that, while the groups were similar in their incorporation of the matcher's descriptors when interacting with the original matcher until round 3, the ASD group became markedly less likely to do so than the NT group when interacting with the *new* matcher on rounds 4 through 5.

**Table 1:** **Incorporation of matcher's descriptors by condition, group and time point**.

		**Same matcher**		**New matcher**	
		***ASD***	***NT***	***ASD***	***NT***
By end of round 3	Participants for whom the matcher suggested descriptors	100% (*n* = 13)	92.3% (*n* = 12)	100%(*n* = 13)	100% (*n* = 13)
	Proportion of the time directors incorporated matcher descriptors M *(SD)*	0.53 (0.38)	0.53 (0.32)	0.44 (0.31)	0.46 (0.25)
Round 4 through 5	Participants for whom the matcher suggested descriptors	38.5% (n = 5)	15.4% (n = 2)	84.6% (n = 11)	100% (n = 13)
	Proportion of the time directors incorporated matcher descriptors M *(SD)*	0.20 (0.18)	0.50 (0.70)	0.18 (0.19)	0.50 (0.27)

*Red indicates cells where data is unreliable because the majority of data was missing*.

## Discussion

Our first set of findings on *referential efficiency* indicate that adults with ASD, who did not have language or intellectual impairment, were similar to a neurotypical comparison group with respect to the initial number of descriptors they used when describing tangram figures, and in the number of exchanges required for a matcher to find the figure they described. They also displayed the typical duration effect observed in lexical entrainment, becoming faster over time, to the same extent as the neurotypical group. These findings indicate that ASD group did entrain on lexical terms in this relatively open-ended task, rather than, for example, perseverating on the same description over five rounds. However, these similarities were observed in the presence of a global delay in completing the game: when directors were adults with ASD the game took significantly longer (on average 36 s longer per round) than when directors were neurotypical adults. This may have been due to differences in variables that we did not measure directly, for example the time taken to formulate descriptions of these complex novel figures, disfluencies when producing the descriptions, and/or the content of the description that may have led to the matcher to respond more slowly although there was no difference in the number of exchanges between director and matcher.

Our second set of findings focused on potential partner-specific effects in round duration and the maintenance of referential precedents. The pattern of results in the neurotypical group showed clear partner-specific effects, where round duration increased dramatically on Round 4 when the *new* matcher was introduced relative to when continuing with the *same* matcher. Interestingly, results from the ASD group belie a strong *impaired theory of mind* account which would predict no difference in round duration across matcher conditions. In fact, the ASD group showed the same condition effect as the neurotypical group, being delayed when the *new* matcher was introduced. Furthermore, there was a marginal interaction indicating that the ASD group had a tendency to be even more delayed by the change in matcher, rather than less delayed as we had expected. With respect to the maintenance of referential precedents, the neurotypical group exhibited robust partner-specific effects again, switching from maintaining the precedent almost all of the time with the *same* matcher to very rarely with the *new* matcher. The ASD group displayed a similar but less pronounced pattern, and were marginally more likely to continue to maintain precedents in critical Round 4 when interacting with a *new* matcher. We also found a counterintuitive correlation in the ASD group between maintenance of precedents on Round 4 when speaking to the new matcher and on duration difference: for those ASD participants who rarely maintained precedents (behaving like the neurotypical group), Round 4 trial duration was significantly longer, leading to negative difference scores. We suggest that this may reflect the effort required by directors with ASD to take the *new* matcher's common ground into account and formulate a more elaborated description as opposed to maintaining the referential precedent they had established with the original, *same* matcher. Importantly, these trends toward differences between groups on Round 4 were amplified on Round 5, where there was a significant interaction whereby the ASD group maintained referential precedents more often than the neurotypical (NT) group when interacting with the *new* matcher.

Data on the *Incorporation of matcher's descriptors* in the new matcher condition allows us to better understand the nature of this effect. Incorporation of descriptors offered by the matcher occurred close to half of the time *when working with the first matcher on Rounds 1 to 3*, among both NT participants and ASD participants. Clark and Wilkes-Gibbs (1986) proposed that when a director feels that the matcher is lacking information, he or she may choose to expand their description prior to being prompted to do so. Directors in our study, whether they were NT or had ASD, did so to a similar degree, incorporating new partner-specific descriptors given feedback that that the previous description was inadequate for the new matcher. This indicates another similarity in partner-specific effects.

However, a significant group difference emerged *when the last two rounds* of the *new* matcher condition were considered. This is the point where the first partner who was involved in lexical entrainment was replaced by a new partner who was viewing the tangrams for the first time. Analyses revealed that NT participants often gave elaborated descriptions on Round 4 when speaking to a *new* matcher (reflecting partner-specific adaptation). In addition, through Round 5, NT directors continued to incorporate terms suggested by the matcher half of the time on average, as they had on earlier rounds with the first matcher. In some cases they abandoned their initial formulation, producing a collaborative referential pact, as in this example, repeated from the methods section:

Participant 117
**Round 4**
  **Director:** a four legged or two legged animal facing the right The head is a parallelogram and its back leg is a rectangle and the front legs look like paws
  ***New* Matcher:** does it look like an elephant if the parallelogram is a trunk?
  **Director:** yeah, it does look like an elephant
**Round 5**
  **Director:** an elephant facing right

Consequently the director in this example did not maintain a referential precedent on Round 5. Sometimes, NT directors did not fully revise their description in favor of a distinct term offered by the matcher, but they added terms the matcher suggested into a collaborative referential pact and the Round 5 description was categorized as same-expanded, for instance:

Participant 124
**Round 3**
  (with ***Same* Matcher**)
  **Director:** lady sitting
**Round 4**
  **Director:** one where the lady is sitting
  ***New* Matcher:** sitting on a regular box?
  **Director**: yes
**Round 5**
  **Director:** the lady sitting on a box

In contrast, ASD participants were less likely to incorporate information offered by the *new* matcher (18% of the time) on Rounds 4 through 5. Instead, ASD participants tended to use the same descriptors they had on Round 4, or a simplification thereof. For example:

Participant 1333
**Round 4**
  **Director:** an arrow as a head
  ***New* Matcher:** is the top a square and then an upside down triangle?
  **Director:** yeah
**Round 5**
  **Director:** an arrow as a head
  ***New* Matcher:** and one foot is in the air?
  **Director**: yeah
Participant 1330
**Round 4**
  **Director:** reading
  ***New* Matcher:** someone's reading?
  **Director:** mmhmm
  ***New* Matcher:** is he upside down?
  **Director:** no it has a triangle on top
  ***New* Matcher:** are there two shapes on either side that are the same? and each of the shapes have 5 sides?
  **Director:** no someone reading facing to the left with a diamond
**Round 5**
  **Director:** the one reading a book

These different patterns of incorporating *new* matcher perspectives on Rounds 4 through 5 in the case of NT directors, and a significantly decreased likelihood to do so by directors with ASD, likely gave rise to the significant interaction for maintenance of a referential pact on Round 5. Yet the groups did not differ with respect to incorporation of the *same* matcher's descriptors on earlier Rounds 1–3. Taken together, these findings demonstrate that adults with ASD do initially incorporate a partner's suggestions to the same degree as NT peers in the context of this task, which once again runs counter to an impaired theory of mind account. However, once a conceptualization and referential precedent has been established (in a collaborative manner), directors with ASD were less flexible in modifying the entrained upon term to accommodate the *new* matcher. Parallel findings were reported by Hala et al. (2007) who found that participants with ASD exhibited normal semantic priming of homographs in a first round of exposure, but not when a prime for the second meaning of the homograph was presented subsequently. Such findings can be explained by difficulties with inhibition or interference control in ASD (e.g., Geurts et al., 2014). Crucially, this is another example of communicative disruption in ASD, customarily attributed to theory of mind impairment, which actually follows from non-social difficulties (e.g., Nadig et al., 2010, where perseverative, self-contingent utterances in conversation were related to restricted and repetitive interest symptoms rather than social skills).

In summary, the adults with ASD in our study displayed largely typical effects of lexical entrainment in a collaborative game requiring them to develop referential descriptions for unlexicalized stimuli, but they took more time to do so than did neurotypical participants matched on verbal IQ. When their partner in the game changed to a *new* matcher, directors with ASD were sensitive to this change as a group, switching from maintaining referential precedents most of the time with the *same* matcher to significantly less often with the *new* matcher.

However, much more variability in making this switch was observed in the ASD than the neurotypical groups, leading to a marginal interaction with a medium effect size. Those directors with ASD who followed the neurotypical pattern of partner-specific adaptation in referential descriptions took significantly longer to complete the round with their partner, suggesting that this adaptation was effortful for them. This was coupled with a significant group difference in incorporating information proposed by the matcher, specifically by the *new* matcher at the end of the game. Neurotypical directors often added elements suggested by the *new* matcher, directors with ASD were resistant to do so at this point when a referential precedent was already established. Potentially due to this, on the last round of the game there was a significant interaction whereby directors with ASD maintained referential precedents with *new* matchers more often than did neurotypical directors. Collectively these represent a range of subtle but likely consequential differences in conversation that should be more pronounced in the context of real-life situations that are less predictable than this game.

We take these findings to indicate that adults with ASD have qualitatively typical patterns of basic lexical entrainment (as reported by Slocombe et al., 2013, for familiar, lexicalized stimuli), but take longer in this process. This global resemblance is offset by multifaceted differences in partner-specific aspects of reference, driven by a subgroup of participants with ASD. Interestingly, differences surfaced in two ways: a minority (4/13) of directors with ASD continued to maintain referential precedents when speaking to a *new* matcher who did not share history with it, while the majority of directors with ASD (9/13) made their descriptions more informative like neurotypical directors, but experienced delays in doing so. It seems that only the minority did not take notice of the *new* matcher's lack of common ground with the referential precedent, while the majority did notice but struggled to produce a description appropriate for their addressee.

We now return to the question of mechanisms that give rise to partner-specific effects in the use of referential pacts, and the debate between “cooperative” views where considerations of common ground guide language use (Clark and Wilkes-Gibbs, 1986; Brennan and Clark, 1996; Metzing and Brennan, 2003; Brown-Schmidt, 2009) and low-level priming and encoding cue views where initial stages of language processing are independent of considerations of common ground (Horton and Gerrig, 2005; Kronmüller and Barr, 2007; Shintel and Keysar, 2007). Our study was not designed to address this debate, but we did find partner-specific effects on production at the first point possible (Round 4) after common ground was manipulated, consistently in neurotypical adults and in the majority of adults with ASD we tested. It is likely that both types of mechanisms are deployed in language use, simultaneously, in a graded fashion depending on a range of relevant factors including the strength of communicative goals, amount of cognitive load, and nature of stimuli (i.e., how entrenched linguistic forms are), (Brennan and Hanna, 2009; Branigan et al., 2011), in line with constraint-based models of language processing (MacDonald et al., 1994; Trueswell and Tanenhaus, 1994). The production paradigm we used pulls for cooperation, or at least mutually comprehensible reference, as there was no default way to describe the figures so descriptions were formulated over time through interaction (see excerpts above). Unlike comprehension studies using familiar, lexicalized stimuli where partner-specific effects can be explained by an expectation for speakers to be referentially consistent (e.g., Shintel and Keysar, 2007), it is difficult to imagine an explanation of our findings that does not entail considerations of the knowledge of the common ground available to the matcher.

A limitation of our study is its small sample size which resulted in some medium size effects not reaching significance. This is balanced by strengths in the rigorous matching of participants across groups and time intensive transcription and detailed coding required for task examining the evolution in production of lexical descriptions over time in a collaborative task. We investigated lexical entrainment between participants and experimental confederates given the logistical constraints of the partner manipulation, among others; an important direction for future work will be to examine lexical entrainment in ASD with naïve participants in both roles, as recommended by Kuhlen and Brennan (2013). Finally, we focused primarily on the director's contributions in this dyadic task; there remain many aspects of the matcher's influence on entrainment to be investigated. Matchers in this task were blind to the hypotheses of the study but not necessarily to group status, which may have become apparent through interaction, though it was not face to face. This gives rise to the possibility that confederate matchers may have engaged in audience design and communicative scaffolding, related to evaluations of the communicative competence (Bortfeld and Brennan, 1997; Branigan et al., 2011) of directors with ASD, which in turn contributed to some of the similarities observed between groups. Though possible we find this unlikely as the confederates were matchers in this game, as opposed to directors who took the lead in formulating descriptions, and the matcher had genuine informational needs as the terms used to describe the complex novel figures were highly idiosyncratic.

Crucially, this first investigation of partner-specific referential pacts in ASD resulted in a complex pattern of results that does not support a categorically *impaired theory of mind* account. The current findings reflect the communicative capacities of adults with ASD who do not have language or intellectual impairment; more pronounced group differences would be expected in children and more representative samples including individuals with ASD who have language and intellectual delays. Future work should explore the nature of two different patterns of partner-specific effects observed here in adults with ASD: not modifying descriptions in the presence of a *new* matcher who did not share common ground in a minority of participants, and adapting descriptions but this entailing a delay in the majority of participants. Further investigation is needed to examine the impact these relatively subtle differences on communication in the lives of people with ASD, including how they are perceived by various conversational partners.

## Funding

This study was funded by a grant from the Max Bell Foundation to the first author (co-PIs AN and Tara Flanagan).

### Conflict of interest statement

The authors declare that the research was conducted in the absence of any commercial or financial relationships that could be construed as a potential conflict of interest.

## Footnotes

^1^Variables presented here are considered to be constructs measuring referential efficiency (especially in the *same* matcher condition). However, given the partner manipulation in the experimental design, effects of condition (where differences are observed between *same* and *new* matcher) are best viewed as partner-specific effects that arose in Rounds 4 and 5 post partner switch.
